# American View of His Work

**Published:** 1920-08-28

**Authors:** 


### THE LATE SIR HENRY BURDETT.

### American View of his Work.

The August number of *The Modern Hospital*, which has just reached us, contains an article upon our late Editor-in-Chief by Dr. Henry M. Hurd, late superintendent of the Johns Hopkins Hospital, Baltimore. To the general remarks made in an editorial in the July number, Dr. Hurd adds a more personal view. The two men were of the same generation and saw the same changes in the hospital world, which made them mutually acquainted. After mentioning the biographical facts Dr. Hurd says:

"Sir Henry Burdett displayed wide and varied activities in constructive philanthropy and rare ability to co-ordinate the energies of others and to secure the assistance of all classes of people in support of his work. He had a talent for friendship, and was well known and appreciated at home in England and Scotland, on the Continent, and in the United States and Canada. He travelled extensively and made friends everywhere. His numerous publications and his technical work in philanthropic and financial matters made him an international authority, and his advice and counsel were greatly sought and generally followed. He will be greatly missed by his friends. Strong and positive were his views as to the need of personal service on the part of all philanthropists, and he constantly urged the duty of such service. His benevolent enterprises were not pursued at the expense of his regular business undertakings—in fact, he became interested in many important business enterprises, and was a useful and active factor in the business life of London. His warm-heartedness prompted his intense interest in all movements calculated to improve the condition of the poor, the sick, and the needy; this quality, together with his unusual ability, made him many warm friends in America as well as in Great Britain. Through his visits to Boston, New York, Philadelphia, Baltimore, and Washington he was well known in these cities, and was intimately associated with the late Dr. John S. Billings in Washington, and with Cowles, Rowe, and Howard of Boston, Fisher and Goldwater of New York, and Weir Mitchell of Philadelphia, and with hospital managers generally. He attended two meetings of the American Hospital Association, one in Boston, and the other in Philadelphia, and had he not been prevented by the Great War, undoubtedly would have revisited America during the past two years.* He was an intense worker and had a clear vision of the future in many departments of the hospital and nursing service. He was also sincerely and devoutly religious, and pursued his work from convictions of responsibility and duty not only to his fellow-men but to his Maker. . . . All who knew him feel his loss deeply, and fear that no one can fill the vacant place."

* Sir Henry Burdett's last visit to the United States was in the autumn of 1916 when he attended the American Hospital Association meeting in Philadelphia.—Ed. *T.H.*

